# 3D Printing Experimental Validation of the Finite Element Analysis of the Maxillofacial Model

**DOI:** 10.3389/fbioe.2021.694140

**Published:** 2021-07-15

**Authors:** Jingheng Shu, Haotian Luo, Yuanli Zhang, Zhan Liu

**Affiliations:** ^1^Key Lab for Biomechanical Engineering of Sichuan Province, Sichuan University, Chengdu, China; ^2^Yibin Institute of Industrial Technology, Sichuan University Yibin Park, Yibin, China; ^3^Department of Medical Technology, Chongqing Three Gorges Medical College, Chongqing, China

**Keywords:** finite element analysis, three-dimensional printing, experimental validation, temporomandibular joint, maxillofacial system

## Abstract

Contacts used in finite element (FE) models were considered as the best simulation for interactions in the temporomandibular joint (TMJ). However, the precision of simulations should be validated through experiments. Three-dimensional (3D) printing models with the high geometric and loading similarities of the individuals were used in the validation. This study aimed to validate the FE models of the TMJ using 3D printing models. Five asymptomatic subjects were recruited in this study. 3D models of mandible, disc, and maxilla were reconstructed according to cone-beam CT (CBCT) image data. PLA was chosen for 3D printing models from bottom to top. Five pressure forces corresponding to the central occlusion were applied to the 3D printing models. Ten strain rosettes were distributed on the mandible to record the horizontal and vertical strains. Contact was used in the FE models with the same geometries, material properties, loadings, and boundary conditions as 3D printing models to simulate the interaction of the disc-condyle, disc-temporal bone, and upper-lower dentition. The differences of the simulated and experimental results for each sample were less than 5% (maximum 4.92%) under all five loadings. In conclusion, it was accurate to use contact to simulate the interactions in TMJs and upper-lower dentition.

## Introduction

Temporomandibular joints (TMJs), which connect the mandible to the skull (glenoid fossa), are a pair of highly complex and mobile joints, with more than 2,000 movements each day during chewing, biting, swallowing, talking, and snoring (Mahdian et al., 2013). During daily activities, TMJs always facilitate mandibular movement by distributing loads to reduce peak stresses. Meanwhile, TMJ is a load-bearing joint where forces are transmitted during mastication (Tanaka and van Eijden, 2003; Poluha et al., 2019). Therefore, the biomechanics of TMJ have to be involved in the study of oral functions.

The finite element (FE) method is a powerful tool to analyze complex systems (i.e., TMJ). The FE models improve the understanding of the behavior of TMJ at different stages of life and with various functional loads (DeVocht et al., 2001; Gregolin et al., 2017; Shu et al., 2020a,b). The simulation also provided the biomechanical properties of TMJ with loading (Beek et al., 2000, 2001b; Sun et al., 2015). In addition, different interaction properties between the disc and the condyle and between the disc and the fossa-eminence complex, including elements that are bonded together and gap elements, were compared and it manifested the contact of a normal TMJ status (Liu et al., 2008, 2016). Finite sliding was allowed between the disc and the condyle, and between the disc and the fossa-eminence complex (Chen et al., 1998). However, bold applications of these simulations in TMJ should be carefully used without precious validation for complex structures. A slight error may lose the reality for the interaction of TMJ. Therefore, the simulated results have to be validated with experiments before drawing any conclusions.

At present, the experimental studies on the biomechanics of TMJ were mainly based on human cadavers and animals. Most of the related experimental studies have been limited to analyze the mechanical properties of TMJs (Kang et al., 1998; Clason et al., 2004; Chen et al., 2016; Cota et al., 2019). The elastic modulus, failure strength, and energy absorption of the disc (Beek et al., 2001a; Kang et al., 2006) and the tensile, compressive, and failure strengths of the mandible (de Zee et al., 2007) were measured by the mechanical experiments of specimens of a human cadaver. In addition, animal experiments were used in the comparison of intraoral vertical ramus osteotomy and sagittal split ramus osteotomy (Zhao et al., 2007). These studies contributed to the mechanical understanding of the TMJ and provided theoretical supports to the simulation and clinics.

Temporomandibular joint-related experimental validations were reported in the previous studies (Gröning et al., 2009; Ramos et al., 2010; Merema et al., 2021). The mandibular geometry used in the FE models was validated by the cadaveric mandible (Ramos et al., 2017). The digital speckle pattern interferometry was conducted to measure the strains on the mandible, and it confirmed the accuracy of the simulation (Gröning et al., 2009). In addition, a polymeric replica of a human mandible was used to validate the FE models to design subsequent artificial TMJ (Ramos et al., 2010). However, these studies only focused on the mandible without the interaction in the TMJ, which was indispensable in investigating the biomechanics of TMJ. Because it was hard to characterize the internal structures and mechanical properties in mandible and maxilla, three-dimensional (3D) printing models with the high geometric and loading similarities of the individuals were used to validate the FE models. The material properties of the 3D printed model were close to those of the bony structures and assigned to the FE models. Thus, this study aimed to validate the maxillofacial FE models under the simulated central occlusion using the 3D printed experimental models.

## Materials and Methods

### Subjects and Data Acquisition

Five asymptomatic subjects (two female and three male, 29.40 ± 8.32 years old) were identified and recruited by a single oral surgeon from the Affiliated Hospital of Stomatology, Chongqing Medical University, China. This study was approved by the institutional review board (IRB), all subjects have signed informed consents before the experiments, and the asymptomatic subjects chosen have no facial deformity and symptoms of temporomandibular disorder (TMD).

The complete head images were scanned by a cone-beam CT (CBCT) machine with slice thicknesses of 0.4 mm. All cross-sectional images were taken following a standardized protocol with 400 pixels × 400 pixels (0.4 mm pixel size).

### Three-Dimensional Modeling

**FIGURE 1:**
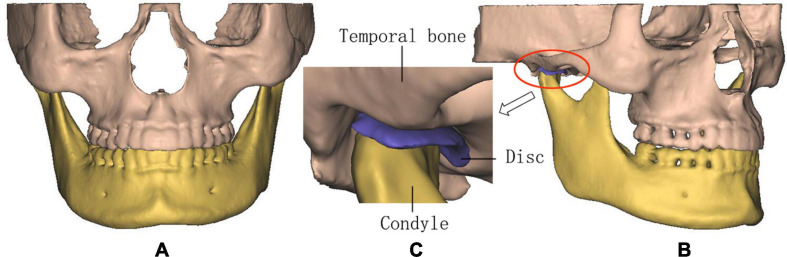
The three-dimensional (3D) finite element (FE) model of temporomandibular joints (TMJs). **(A)** Front view, **(B)** lateral view, and **(C)** details in TMJ.

The CBCT data were transferred to the Digital Imaging and Communications in Medicine (DICOM) format and imported into the Materialise Interactive Medical Image Control System (MIMICS) 15.0 (Materialise, Leuven, Belgium) for model building. The 3D models including the mandible, maxilla, and teeth of all the subjects were constructed based on each slice of CBCT. The articular discs were established according to the anatomical structure and the shape of constructed mandible and maxilla. The above structures of TMJs were saved as surface triangulation technique (STL) format and imported into ABAQUS 6.13 (Dassault SIMULIA, RI) for the generation of the 3D FE models of all the subjects (Figure 1).

**FIGURE 2:**
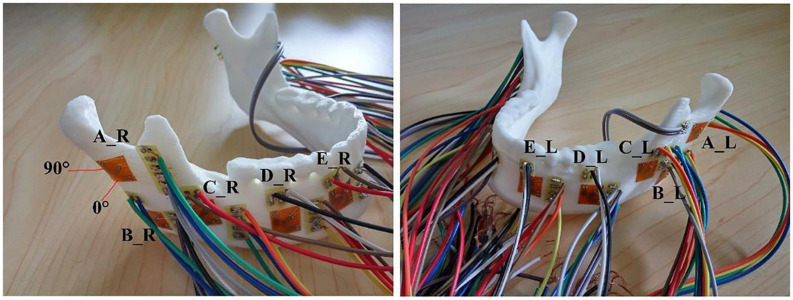
The distributions of 10 strain rosettes on the mandible: A located at the bilateral mandibular condyle necks, B located at the bilateral mandibular angles, and C, D, and E located at the bilateral mandibular bodies. R and L represented the right and left sides, respectively.

The STL files of the models were imported into the 3D printer, and then, the 3D experimental models were obtained (Figure 2). Polylactic acid (PLA) was selected for 3D printing from the bottom to the top due to its good mechanical strength, elastic modulus, thermoforming properties, and mechanical similarity with jawbones (Weng et al., 2000; Kong et al., 2005), because it is difficult to simulate the heterogeneous and orthotropic properties of bony structure without a cadaver. 3D printing models with PLA were useful and were available for the validation of contact simulation in TMJ. The thickness of the printing layer was 0.2 mm with 100% filling, and the printing temperature was 210°C.

### Mechanical Properties Test

Ten PLA 3D printed specimens based on the national standard were used to determine Young’s modulus and Poisson’s ratio. Since the mechanical properties of PLA after 3D printing would change, tensile tests of PLA 3D printed specimens should be carried out. According to the size of the specimens of national standard, the specimens for the tests were constructed. The STL files of the specimens were imported into the same 3D printer with the same conditions of TMJ models. Then, the tensile tests were performed on universal testing machine AG-IS (Shimadzu, Japan) and static strain gauge DH3818 (Donghua, China), and Young’s modulus and Poisson’s ratio were 1.69 ± 0.14 GPa and 0.3 ± 0.05, respectively.

### Simulated Central Occlusion Experiments

**FIGURE 3:**
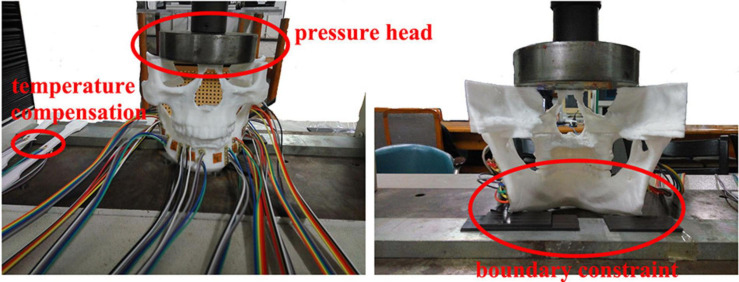
The 3D experimental models of TMJs.

The 10 strain rosettes were distributed on the mandibular condyle necks, the mandibular angles, and the mandibular bodies of the experimental models (Figure 2). The monitoring regions were bilaterally arranged. The vertical pressure forces of 100 N, 150 N, 200 N, 250 N, and 300 N were applied to the experimental models for simulating central occlusion on the universal testing machine AG-IS (Figure 3). The magnitudes of these forces were derived from previous studies, and they were shifted to the top surface as a pressure force to conform to this occlusion (Lin et al., 2006; Shu and Liu, 2020). The constraint regions were marked to provide the simulated constraints.

### Finite Element Analysis

The bone and the discs were all modeled as linear elastic according to the experiments (Shu et al., 2019). The interactions of the disc-condyle, disc-temporal bone, and upper-lower dentition were considered as contact with a frictional coefficient of 0.001. The modified 10-node quadratic tetrahedron element (C3D10M) was used in the contact regions. The four-node linear tetrahedron element (C3D4) was used for the other regions of the models. The total number of elements for all the models was about 170,000. The loading and boundary conditions were identical with the 3D printed models in the experiments. The static solver was used to simulate the quasi-static status of the experiments.

### Comparison and Statistical Analysis

For each model with the same load, the strains between the experimental and FE models were compared at each monitoring point. The difference was expressed as follows:

$$\text{Difference}=\frac{\left|\mathrm{Simulation}-\mathrm{Experiment}\right|}{\text{Expriment}}\times 100\%$$

## Results

**FIGURE 4:**
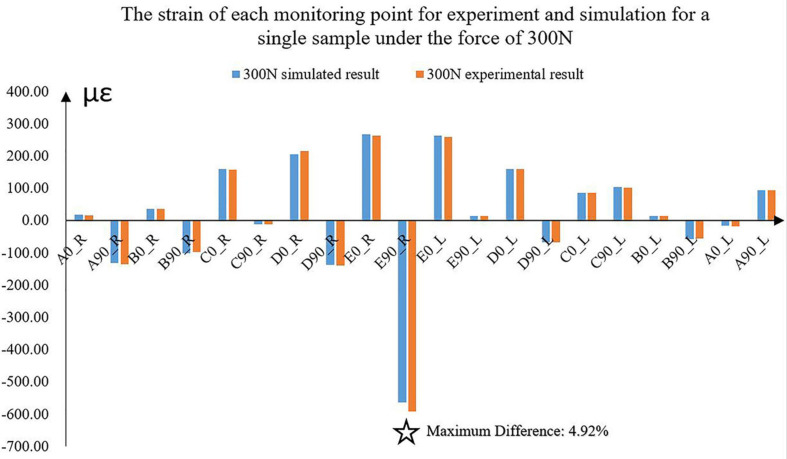
The experimental and simulated strains of the 10 monitoring points for a sample under the force of 300 N. ✩ represented the maximum difference between experimental and simulated strains. A located at the bilateral mandibular condyle necks, B located at the bilateral mandibular angles, and C, D, and E located at the bilateral mandibular bodies. 0 and 90 represented the horizontal and vertical directions, respectively. R and L represented the right and left sides, respectively.

The differences between the simulated and experimental results were compared. With the increase in loadings, the differences between simulated and experimental strains correspondingly increased. The maximum difference was 4.92% under the force of 300 N for one sample (Figure 4). The average strain difference between the simulation and the experiment of this sample under the force of 300 N was 2.43%, which was the maximum among the samples. The average differences of all the other samples were 1.46 and 2.30%. Under the other forces, the differences decreased to 1%.

**FIGURE 5:**
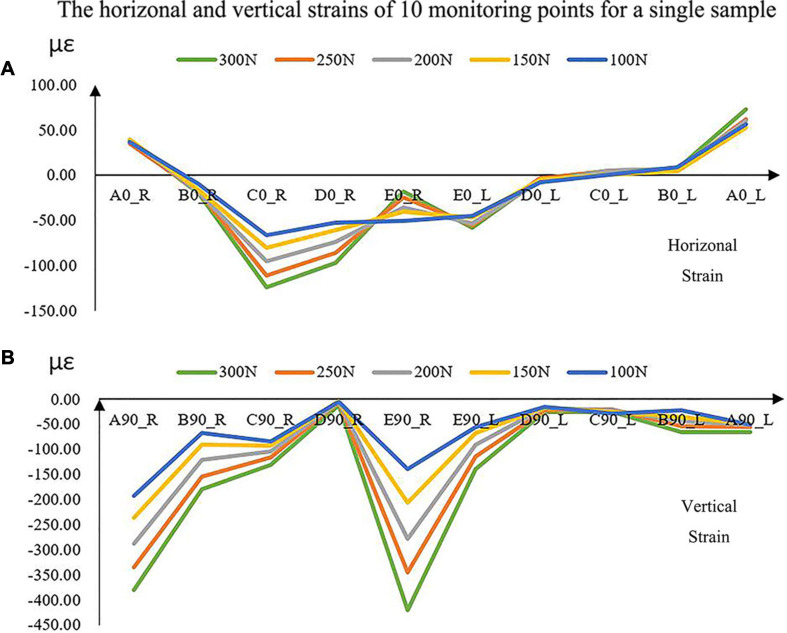
The experimental horizontal **(A)** and vertical **(B)** strains of the 10 monitoring points with increased loads for a single sample. A located at the bilateral mandibular condyle necks, B located at the bilateral mandibular angles, and C, D, and E located at the bilateral mandibular bodies. 0 and 90 represented the horizontal and vertical directions, respectively. R and L represented the right and left sides, respectively.

The magnitudes of all the strains increased with the increase in force (Figure 5). The vertical strains of each monitoring point were all expressed as compressive strains under all forces (Figure 5). However, the horizontal strains located at the bilateral condyle necks were always presented as tensile strains. It was complex at other positions in the horizontal directions due to the individual geometric differences.

**FIGURE 6:**
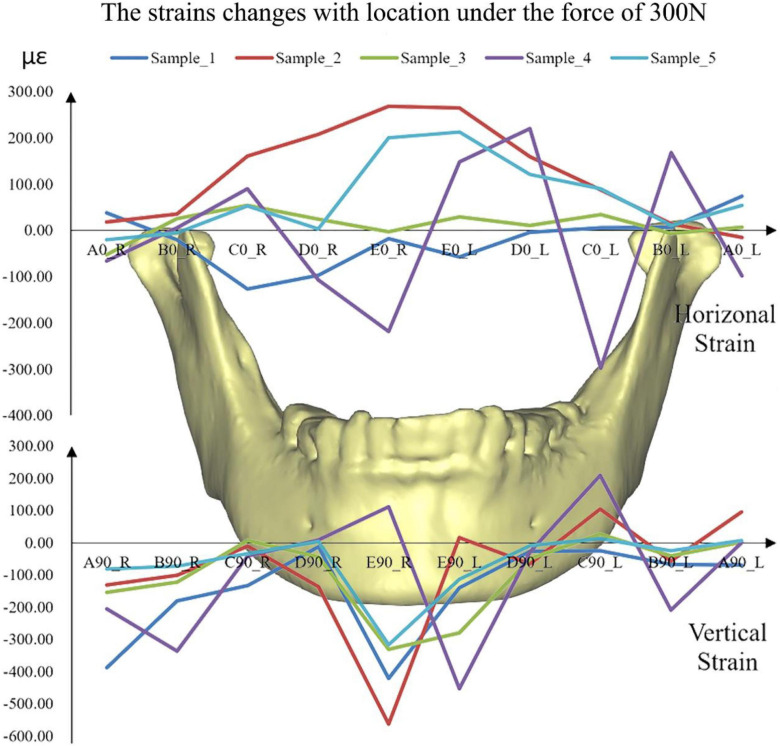
The simulated strains from FE models of all the five samples under the force of 300 N. A located at the bilateral mandibular condyle necks, B located at the bilateral mandibular angles, and C, D, and E located at the bilateral mandibular bodies. 0 and 90 represented the horizontal and vertical directions, respectively. R and L represented the right and left sides, respectively.

The distributions of the strains on the mandible were different from each other (Figure 6). The strains, especially vertical strains, at the mandibular body close to the facial midline (i.e., monitoring point E) were generally greater than those at other locations. In addition, the strains on the mandibular ramus (monitoring point A) were slightly greater than those on the edges of the mandibular body (monitoring point C).

## Discussion

Finite element analysis (FEA) was usually used in the study of oral biomechanics (Dicker et al., 2012; Qi et al., 2012; Liu et al., 2016; Demircan et al., 2020; Lai et al., 2020). Several interactions were considered in the simulation of TMJ (Liu et al., 2008). Contact was proven to be good in the simulation of complex interactions of maxillofacial models (Liu et al., 2008, 2016). However, there was no validation of the precision of contact to simulate the interactions in the TMJs. The cadaver models were not adopted due to the difficulty in determination of the heterogeneity of the geometry and internal bone characteristics. Thus, this study aimed to verify the precision of simulation using the experiments of 3D printing models.

The condition of the simulated models was the same as the experimental samples. 3D printed and FE models shared the same geometry based on the constructed models from medical images. The material properties of the FE models were from the mechanical properties test. Moreover, the loadings and the boundary conditions from the experiments were applied to the corresponding FE models. In addition, the simulated stress distribution of the disc was similar to the centric occlusions (Beek et al., 2000; Shu et al., 2019), with its high stress located at the lateral intermediate zone. The monitoring points for both groups were also the same. Thus, the experimental validation of the maxillofacial system was reliable.

Under the five different pressure forces, the maximum difference of all the samples between simulated and experimental results was 4.92% among all monitoring points (Figure 4). The maximum difference was located at the middle of the mandibular body of Sample 2 (Figure 6). It was clear that the changes in the forces did not affect the differences between experiments and simulations. Furthermore, the vertical strains always presented compressive strains with the increase in forces from 100 to 300 N. The magnitudes of all the strains tended to increase with the increase in forces (Figure 5). Therefore, contact was reasonable to simulate the contact of TMJ.

The rhythmicity of the strains with the positions was different from each other due to the differences in individual geometry of the maxilla and mandible (Figure 6). In general, monitoring point E showed the greatest horizontal and vertical strains. The results showed in mandibular bodies are the closer to the facial midline, the greater the strain. In the vertical direction, monitoring points A (mandibular condyle necks) and B (mandibular angles) presented greater strains compared to points C and D (mandibular bodies).

Three replicate experiments were performed on each sample with the same force. Low deviations were found in the measurements of three replicate experiments. The high repeatability of the experiments was reflected. However, on one hand, the experimental results were influenced by the experimental conditions (humidity and temperature) and instruments. On the other hand, the FE outcomes should be the only results under the same loading condition. Thus, the accurate FE models were more controllable and freer from environmental disturbances.

The combined 3D printing experiments and FEA could be further used in clinics. The biomechanical environment could be evaluated, and then, the individual treatment plan could be designed. It could be also applied in maxillofacial surgery, and the simulation of bilateral sagittal split ramus osteotomy (BSSRO) could be applied to the FE models to evaluate the postoperative biomechanical status of the patients. These postoperative outcomes could also help clinicians optimize surgical strategies and prevent postoperative complications. In addition, the validations of FE models of the TMJ ensured the biomechanical design of the prosthesis of TMJ. In the previous study, a human cadaver was used to validate the correction of condyle implants of humans (Mesnard and Ramos, 2016). In this study, 3D printing experiments could also provide more precious validation of FE models to improve the condylar implants in TMJ.

One major limitation of this study was that all the samples of this study were asymptomatic subjects. The models of patients should be reconstructed to provide further verification. Another limitation was that 3D printing models could not characterize the heterogeneity and orthotropy of human mandible, maxilla, and disc, like previous studies using FE models and cadaver experiments (Ichim et al., 2006; Ramos et al., 2010). However, FE models could be considered as heterogeneous and orthotropic and be proven as accurate within the allowable range of error. Furthermore, although the constraints were marked to ensure the consistency of experiments and simulations, there would be some errors in the constraint.

## Conclusion

The vertical and horizontal strains between the experiments on 3D printed models and the FEA had less than 5% differences for all the samples. It proved that the FE models could provide strains within a minimum 95% agreement. Therefore, it was accurate to use contacts to simulate the interactions in TMJs in future research studies and applications.

## Data Availability Statement

The original contributions presented in the study are included in the article/supplementary material, further inquiries can be directed to the corresponding author/s.

## Ethics Statement

The studies involving human participants were reviewed and approved by Institutional Review Board (IRB) of the Affiliated Hospital o Stomatology, Chongqing Medical University. The patients/participants provided their written informed consent to participate in this study. Written informed consent was obtained from the individual(s) for the publication of any potentially identifiable images or data included in this article.

## Author Contributions

JS provided the ideas, designed a complete research program, analyzed the data of 3D model and FEM model, and was responsible for drafting the manuscript. YZ and HL collected the experimental data and completed the simulation. HL carried the 3D printing experiment according to the CT image data. ZL got the support of the National Natural Science Foundation of China and the Government of Yibin and provided technical guidance for the whole research. All authors contributed to the article and approved the submitted version.

## Conflict of Interest

The authors declare that the research was conducted in the absence of any commercial or financial relationships that could be construed as a potential conflict of interest.

## References

[B1] Beek M. Aarnts M. P. Koolstra J. H. Feilzer A. J. van Eijden T. (2001a). Dynamic properties of the human temporomandibular joint disc. *J. Dental Res.* 80 876–880. 10.1177/00220345010800030601 11379888

[B2] Beek M. Koolstra J. H. van Ruijven L. J. van Eijden T. M. G. J. (2000). Three-dimensional finite element analysis of the human temporomandibular joint disc. *J. Biomech.* 33 307–316. 10.1016/s0021-9290(99)00168-2 10673114

[B3] Beek M. Koolstra J. H. van Ruijven L. J. van Eijden T. M. G. J. (2001b). Three-dimensional finite element analysis of the cartilaginous structures in the human temporomadibular joint. *J. Dental Res.* 80 1913–1918. 10.1177/00220345010800101001 11706951

[B4] Chen J. Akyuz U. Xu L. Pidaparti R. M. (1998). Stress analysis of the human temporomandibular joint. *Med. Eng. Phys.* 20 565–572. 10.1016/s1350-4533(98)00070-8 9888234

[B5] Chen J. N. Suenaga H. Hogg M. Li W. Swain M. Li Q. (2016). Determination of oral mucosal Poisson’s ratio and coefficient of friction from in-vivo contact pressure measurements. *Comput. Methods Biomech. Biomed. Eng.* 19 357–365. 10.1080/10255842.2015.1028925 26024011

[B6] Clason C. Hinz A. M. Schieferstein H. (2004). A method for material parameter determination for the human mandible based on simulation and experiment. *Comput. Methods Biomech. Biomed. Eng.* 7 265–276. 10.1080/10255840412331313590 15621649

[B7] Cota J. M. G. Leale D. M. Arzi B. Cissell D. D. (2019). Regional and disease-related differences in properties of the equine temporomandibular joint disc. *J. Biomech.* 82 54–61. 10.1016/j.jbiomech.2018.10.017 30392775

[B8] de Zee M. Dalstra M. Cattaneo P. M. Rasmussen J. Svensson P. Melsen B. (2007). Validation of a musculo-skeletal model of the mandible and its application to mandibular distraction osteogenesis. *J. Biomech.* 40 1192–1201. 10.1016/j.jbiomech.2006.06.024 16930608

[B9] Demircan S. Ureturk E. U. Apaydin A. Sen S. (2020). Fixation methods for mandibular advancement and their effects on temporomandibular joint: a finite element analysis study. *Biomed. Res. Int.* 2020:8. 10.1155/2020/2810763 PMC7060428 32185199

[B10] DeVocht J. W. Goel V. K. Zeitler D. L. Lew D. (2001). Experimental validation of a finite element model of the temporomandibular joint. *J. Ora Maxillofac. Surg.* 59 775–778. 10.1053/joms.2001.24292 11429739

[B11] Dicker G. J. Tuijt M. Koolstra J. H. Van Schijndel R. A. Castelijns J. A. Tuinzing D. B. (2012). Static and dynamic loading of mandibular condyles and their positional changes after bilateral sagittal split advancement osteotomies. *Int. J. Oral maxillofac. Surg.* 41 1131–1136. 10.1016/j.ijom.2012.03.013 22525894

[B12] Gregolin R. F. Zavaglia C. A. D. Tokimatsu R. C. Pereira J. A. (2017). Biomechanical stress and strain analysis of mandibular human region from computed tomography to custom implant development. *Adv. Mater. Sci. Eng.* 2017:9.

[B13] Gröning F. Liu J. Fagan M. J. O’Higgins P. (2009). Validating a voxel-based finite element model of a human mandible using digital speckle pattern interferometry. *J. Biomech.* 42 1224–1229. 10.1016/j.jbiomech.2009.03.025 19394021

[B14] Ichim I. Swain M. Kieser J. A. (2006). Mandibular biomechanics and development of the human chin. *J. Dental Res.* 85 638–642. 10.1177/154405910608500711 16798865

[B15] Kang H. Bao G. J. Qi S. N. (2006). Biomechanical responses of human temporomandibular joint disc under tension and compression. *Int. J. Oral Maxillofac. Surg.* 35 817–821. 10.1016/j.ijom.2006.03.005 16697140

[B16] Kang H. Yi X. Z. Chen M. S. (1998). A study of tensile mechanical property of human temporomandibular joint disc. *West China J. Stomatol.* 16 253–255.

[B17] Kong H. Pan K. F. Zhang S. J. (2005). The study of the new modified copolymer of PLA and PEG on the repair of rabbit mandibular defect. *J. Oral Maxillofac. Surg.* 15 24–28.

[B18] Lai L. F. Huang C. Y. Zhou F. Xia F. J. Xiong G. F. (2020). Finite elements analysis of the temporomandibular joint disc in patients with intra-articular disorders. *BMC Oral Health* 20:8. 10.1186/s12903-020-01074-x 32228551 PMC7106847

[B19] Lin C. L. Wang J. C. Kuo Y. C. (2006). Numerical simulation on the biomechanical interactions of tooth/implant-supported system under various occlusal forces with rigid/non-rigid connections. *J. Biomech.* 39 453–463. 10.1016/j.jbiomech.2004.12.020 16389085

[B20] Liu Z. Fan Y. Qian Y. (2008). Comparative evaluation on three-dimensional finite element models of the temporomandibular joint. *Clin. Biomech.* 23 Suppl 1 S53–S58. 10.1016/j.clinbiomech.2007.12.011 18282646

[B21] Liu Z. Qian Y. L. Zhang Y. L. Fan Y. B. (2016). Effects of several temporomandibular disorders on the stress distributions of temporomandibular joint: a finite element analysis. *Comput. Methods Biomech. Biomed. Eng.* 19 137–143. 10.1080/10255842.2014.996876 25587737

[B22] Mahdian N. Dostalova T. J. Danek J. Nedoma J. Kohout J. Hubacek M. (2013). 3D reconstruction of TMJ after resection of the cyst and the stress-strain analyses. *Comput. Methods Programs Biomed.* 110 279–289. 10.1016/j.cmpb.2012.12.001 23332173

[B23] Merema B. B. J. Kraeima J. Glas H. H. Spijkervet F. K. L. Witjes M. J. H. (2021). Patient-specific finite element models of the human mandible: lack of consensus on current set-ups. *Oral Dis.* 27 42–51. 10.1111/odi.13381 32372548 PMC7818111

[B24] Mesnard M. Ramos A. (2016). Experimental and numerical predictions of Biomet((R)) alloplastic implant in a cadaveric mandibular ramus. *J. Craniomaxillofac. surg.* 44 608–615. 10.1016/j.jcms.2016.02.004 27017105

[B25] Poluha R. L. Canales G. T. Costa Y. M. Grossmann E. Bonjardim L. R. Conti P. C. R. (2019). Temporomandibular joint disc displacement with reduction: a review of mechanisms and clinical presentation. *J. Appl. Oral Sci.* 27:e20180433. 30810641 10.1590/1678-7757-2018-0433 PMC6382319

[B26] Qi X. D. Ma L. M. Zhong S. Z. (2012). The influence of the closing and opening muscle groups of jaw condyle biomechanics after mandible bilateral sagittal split ramus osteotomy. *J. Craniomaxillofac. Surg.* 40 e159–e164. 21907586 10.1016/j.jcms.2011.07.024

[B27] Ramos A. Ballu A. Mesnard M. Talaia P. Simões J. A. (2010). Numerical and experimental models of the mandible. *Exp. Mech.* 51 1053–1059. 10.1007/s11340-010-9403-x

[B28] Ramos A. Nyashin Y. Mesnard M. (2017). Influences of geometrical and mechanical properties of bone tissues in mandible behaviour - experimental and numerical predictions. *Comput. Methods Biomech. Biomed. Eng.* 20 1004–1014. 10.1080/10255842.2017.1322072 28446031

[B29] Shu J. Liu Z. (2020). The Biomechanical comparisons of different periodontal conditions under the different extracoronal precision attachment restorations for the mandibular kennedy i dentition defect. *J. Mech. Med. Biol.* 20:2050019. 10.1142/s0219519420500190

[B30] Shu J. Ma H. Jia L. Fang H. Chong D. Y. R. Zheng T. (2020a). Biomechanical behaviour of temporomandibular joints during opening and closing of the mouth: a 3D finite element analysis. *Int. J. Numer. Methods Biomed. Eng.* 36:e3373. 10.1002/cnm.3373 32453468

[B31] Shu J. Teng H. Shao B. Zheng T. Liu Y. Liu Z. (2020b). Biomechanical responses of temporomandibular joints during the lateral protrusions: a 3D finite element study. *Comput. Methods Programs Biomed.* 195:105671. 10.1016/j.cmpb.2020.105671 32721783

[B32] Shu J. Zhang Y. Liu Z. (2019). Biomechanical comparison of temporomandibular joints after orthognathic surgery before and after design optimization. *Med. Eng. Phys.* 68 11–16. 10.1016/j.medengphy.2019.03.018 30979582

[B33] Sun M. X. Yang J. J. Zhou R. Z. Li N. Y. Xia J. N. Gu F. (2015). Mechanical analysis on individualized finite element of temporal-mandibular joint under overlarge jaw opening status. *Int. J. Clin. Exp. Med.* 8 9046–9054. 26309558 PMC4538150

[B34] Tanaka E. van Eijden T. (2003). Biomechanical behavior of the temporomandibular joint disc. *Crit. Rev. Oral Biol. Med.* 14 138–150. 10.1177/154411130301400207 12764076

[B35] Weng Y. L. Cao Y. L. Vacanti C. A. (2000). The experimental study of tissue engineered mandible condyle in the shape of human. *Shanghai J. Stomatol.* 9 94–96. 15014818

[B36] Zhao Q. Hu J. Wang D. Zhu S. (2007). Changes in the temporomandibular joint after mandibular setback surgery in monkeys: intraoral vertical versus sagittal split ramus osteotomy. *Oral Surg. Oral Med. Oral Pathol. Oral Radiol. Endod.* 104 329–337. 10.1016/j.tripleo.2006.12.024 17428700

